# Assessment of exposure to sexually explicit materials and factors associated with exposure among preparatory school youths in Hawassa City, Southern Ethiopia: a cross-sectional institution based survey

**DOI:** 10.1186/s12978-015-0068-x

**Published:** 2015-09-14

**Authors:** Tony Habesha, Zewdie Aderaw, Serawit Lakew

**Affiliations:** Welayta Zone Health Department, SNNPR Health Bureau, Ministry of Health, Addis Ababa, Ethiopia; Department of Nursing and Midwifery, Arba Minch College of Health Sciences, Arba Minch, Southern Ethiopia Ethiopia; Department of Public Health, Debre Markos University, Debre Marqos, Northern Ethiopia Ethiopia

## Abstract

**Background:**

According to the 2007 Ethiopian census, youths aged 15–24 years were more than 15.2 million which contributes to 20.6 % of the whole population. These very large and productive groups of the population are exposed to various sexual and reproductive health risks. The aim of this study was to assess exposure to Sexually Explicit Materials (SEM) and factors associated with exposure among preparatory school students in Hawassa city, Southern Ethiopia.

**Methodology:**

A cross-sectional institution based study involving 770 randomly selected youth students of preparatory schools at Hawassa city. Multi stage sampling technique was used to select study subjects. Data was collected using pre-tested and self-administered questionnaire. Data was entered by EPI INFO version 3.5.1 and analyzed using SPSS version 20.0 statistical software packages. The result was displayed using descriptive, bivariate and multivariate analysis. Statistical association was done for independent predictors (at *p* < 0.05).

**Result and discussion:**

About 750 students were participated in this study with a response rate of 97.4 %. Among this, about 77.3 % of students knew about the presence of SEM and most of the respondents 566(75.5 %) were watched SEM films/movies and 554(73.9 %) were exposed to SE texts. The overall exposure to SEM in school youths was 579(77.2 %). Among the total respondents, about 522(70.4 %) claimed as having no open discussion on sexual issues with in their family. Furthermore, About 450 (60.0 %) respondents complained for having no sexual and reproductive health education at their school. Male students had faced almost two times higher exposure to SEM than female students (95 % CI: AOR 1.84(C.I = 1.22, 2.78). Students who attended private school were more than two times more likely exposed to SEM than public schools (95 % CI: AOR 2.07(C.I = 1.29, 3.30). Students who drink alcohol and labelled as ‘sometimes’ were two times more likely exposed to SEM than those who never drink alcohol (95 % CI = AOR 2.33(C.I = 1.26, 4.30). Khat chewers who labelled “rarely”, “sometimes” and “often” had shown higher exposure (95 % CI: AOR 3.02(CI = 1.65, 5.52), (95 % CI: AOR 3.40(CI = 1.93, 6.00) and (95 % CI: AOR 2.67(CI = 1.46, 4.86) than those who never chew khat, respectively. Regarding SEM access, school youths with label ‘easy access were exposed in odds of six folds than youths of no access (95 % CI: AOR 5.64(C.I = 3.56, 8.9).

**Conclusion:**

High number of students was exposed to sexually explicit materials. Sex, school type, substance use and access to SEM were observed independent predictors of exposure to SEM.

**Motivation:**

The current generation of young people is the healthiest, most educated, and most urbanized in history. However, there still remain some serious concerns. Most people become sexually active during adolescence. Premarital sexual activity is common and is on the rise worldwide. Rates are highest in sub Saharan Africa, where more than half of girls aged 15–19 are sexually experienced. Millions of adolescents are bearing children, in sub-Saharan Africa. More than half of women give birth before age 20. The need for improved health and social services aimed at adolescents, including reproductive health services, is being increasingly recognized throughout the world. Approximately 85 % of world adolescents live in developing countries. Each year, up to 100 million becomes infected with a curable sexually transmitted disease (STI). About 40 % of all new global human immunodeficiency virus (HIV) infections occur among 15–24 year olds; with recent estimates of 7000 infected each day. These health risks are influenced by many interrelated factors, such as expectations concerning early marriage and sexual relationships, access to education and employment, gender inequities, sexual violence, and the influence of mass media and popular culture. Furthermore, many adolescents lack strong stable relationships with parents or other adults whom they can talk to about their reproductive health concerns. Despite these challenges, programs that meet the information and service needs of adolescents can make a real difference. Successful programs help young people develop life-planning skill, respect the needs and concerns of young people, involve communities in their efforts, and provide respectful and confidential clinical services. Accordingly, the government of Ethiopia now works on improving adolescent’s health as one part of MDG (Goal VI-halting transmission of HIV/AIDS, STI, and other communicable diseases) with a focus on adolescents, since they are most affected population. This finding, therefore, will benefit the government to partly evaluate the goal achieving through adolescents exposure status to sexually explicit materials and improvement of sexual issues free talk with in school with class mates and their family at home. For that matter, we authors decided to publish this finding in BMC Reproductive Health Journal so that on line access will be easy to all governing bodies that they use to re-plan their strategies for better product of plan. Moreover, Researchers, Practitioners, policy makers, Students, school leaders and professionals will also benefit from this finding for their future researches references, knowledge gain and practice.

## Background

More than one billion people in the world are between the ages of 15 and 24. Most of these live in developing countries [1]. In Ethiopia, youths aged 15–24 years were more than 15.2 million, contributing to 20.6 % of the whole population [2]. These large and productive groups of the population are exposed to various sexual and reproductive health risks. Among many sexual and reproductive health risks: sexual coercion, early marriage, polygamy, female genital cutting, unplanned pregnancies, closely spaced pregnancies, abortion, and sexually transmitted infections (STIs) are the major ones [1].

Various studies showed that males have been found to be more likely to expose themselves to SEMs than females (Such as, 7 times likely to report online seeking (*p < 0*.001) and 4 times likely to report offline-only seeking (*p < 0*.001)) [3–5]. Girls are more likely than boys to be troubled by sexually explicit images. Thirty five percent of girls but only six per cent of boys reported that they were very upset by the experience [6, 7].

As one study in USA suggested, youths 14 years and older were almost three times likely to report online seeking behavior compared to younger youths (*p <* 0.001). No significant differences in age were noted between youth who reported offline-only seeking and non-seeking behavior. All Internet usage characteristics failed to significantly differentiate reports of pornography seeking behavior [4].

Various studies outside home observed that older adolescents tend to view sexual contents online more often than younger internet users. Higher religiosity is linked to delays in sexual development. Lower religiosity is linked to greater exposure to online sexual materials [3, 4, 8].

Studies of New Hampshire identified parental Internet controls. None of its four measures were significantly differentiated youth by their self-report of pornography seeking behavior. Similarly high percentages (85–93 %) of caregivers reported a household rule about disallowing Internet pornography sites across the three groups of young people. When asked whether a filter or blocking software was installed on the computer, 27 % of caregivers and 16 % of youth online seekers, versus 22 % of caregivers and 19 % of youth offline seekers, and 23 % of both caregivers and youth non-seekers responded positively [4].

North Carolina State of USA finding suggested that sexual risk behavior among young people demonstrated that quality of the parent—child relationship, parent—child communication, and peer support represent interacting social systems that are related to sexual risk behavior. Young people who report higher levels of connectedness with parents have lower rates of unprotected sexual intercourse, engage in sexual intercourse with fewer partners, older at first sexual intercourse and make safer sexual decisions [9]. In eastern Michigan and other study findings, young people living in intact families are more likely to delay sexual activity and report less sexual experience than peers living in other family forms. Parents’ previous sexual experiences were not significantly related to parent-teen communication, but more information is needed in order to determine the specific relation to these conversations [10, 11]. In home study, Daily Khat intake was also associated with unprotected sex. There was a significant and linear association between alcohol intake and unprotected sex, with those using alcohol daily having a three fold increased odds compared to those not using it. Use of substances other than Khat was not associated with unprotected sex, but was associated with initiation of sexual activity [12].

The caregiver-child relationship was an important influence in estimating the likelihood of reporting pornography exposure. Youth who reported a poor emotional bond with their caregiver were twice as likely also to report online-seeking behavior compared to a similar group youth who reported a strong emotional bond (*p < 0*.01). Frequent coercive discipline was significantly related to 67 % higher adjusted conditional odds of reporting offline-only seeking behavior versus non-seeking behavior (*p < 0*.05). Delinquent behavior was associated with a 4-fold increase in adjusted conditional odds of reporting either online-seeking behavior (*p < 0*.001) or offline-only seeking behavior (*p < 0*.001) compared to non-seeking behavior after adjusting for all other influential characteristics, findings of New Hampshire National Survey [4]. Delinquent youth not only are more likely to have been exposed to pornography but also report more exposure, exposure at an earlier age (often under 10), and more extreme pornography use than their peers [13].

New Hampshire, USA, study also found that Substance use was related to more than a two-fold increase in adjusted conditional odds in disclosing online (*p < 0*.001) as well as offline-only (*p < 0*.01) seeking behavior compared to similar youth who reported negligible substance use. Young people who reported unintentional exposure to sexual material online were more than 2.5 times likely to report intentional exposure online compared to similar young people who did not report unintentional exposure (*p < 0*.001) [4].

Youth in the United States and increasingly around the world spend more time with the media than they do in school or with their parents [14, 15]. Much of what young people are listening to and/or watching includes sexual content, but, unfortunately, very little that might be considered sexually healthy [16]. Adolescents with predominantly older friends may be confronted more often with people with more elaborate sexual experiences; and with younger friends may meet more frequently people with less elaborate sexual experiences [17]. High speed internet connections also allow access to a relatively large amount of data in a short time, which consequently may influence the amount of viewed sexual images [18].

## Methods and materials

### Study design, study area and period

A cross-sectional study design was employed on randomly selected preparatory school students of the Hawassa City. The study was conducted in Hawassa city, which is a capital of South Ethiopia Regional State, located some 275 km from Addis Ababa. Currently, there are 10 preparatory schools (2 public and 8 private). From a total of 6245 students, about 2825 were females [19]. The city is dominated by Sidama, Wolaita, Amhara, Guraghe and Oromo ethnic groups and the official language is Amharic. The town has eight subcity administrative zones and access to broadband internet services (such as, Wi-Fi). The study was held from May 1 to May 12/2014.

### Sampling procedure and sample size determination

**Fig 1:**
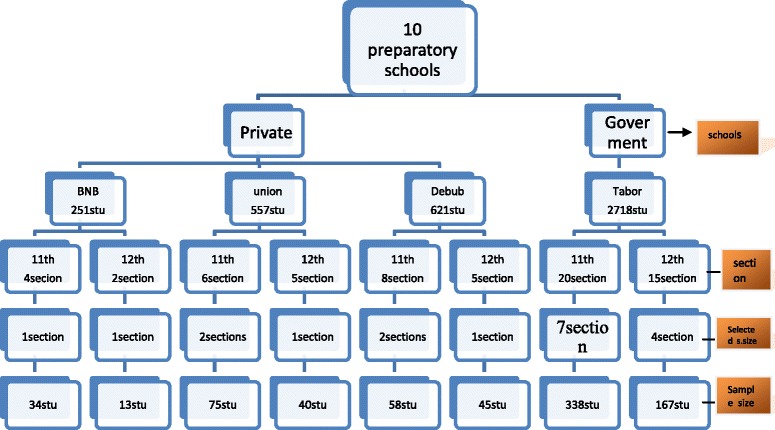
Schematic presentation of sampling procedure

To determine the sample size for the study population the following steps were used. Formula for single Population proportion was used. Assumptions for 5 % marginal error (d) and 95 % confidence interval (α =0.05) used. Estimated prevalence of textual exposure obtained from previous study was *p* = 0.65. Accordingly, total sample size was 770. For the selection of these respondents, multi-stage sampling technique was employed. There were ten preparatory schools in Hawassa city, two were public and eight were private schools. One public and three private schools were selected using simple random sampling technique. For the four schools, respondents were allocated using Population proportionate to size (PPS) technique. Here, student’s roster (list) was used as sampling frame. In each of these schools, students were assigned as Grade 11 and 12. From these grades, sections of students were selected by lottery method. Participants in each of selected section of students were selected by lottery method (using students’ attendance sheet). Figure 1 sampling procedure.

### Data collection and data quality assurance

Data were collected using respondent administered questionnaire. A questionnaire consisted of 60 variables, which was categorized into three parts. This includes Socio—demographic, personal characteristics and other exposure variables. Each variable had list of responses to be responded only by the participant. To assure the data quality, 2 days training was given to four data collectors and two supervisors. Appropriate information and instruction on the objective and relevance of the study was given to the respondents. The data collectors were stayed with respondents until all questions filled and responded. Informed consent was also secured to the respondents.

### Data management and data analysis

After data collection, each questionnaire was checked for completeness, consistency, and clarity and entered into the template and re-checked for errors. Data entry was done using EPI info version 3.5.1 statistical software and exported to SPSS windows version 16 for further processing and analysis. Attitude questions were summed and a mean score was calculated to categorize the overall attitude of the respondents. Bivariate analysis using binary logistic regression model was used to determine the association between independent predictors.

Variables found to be associated in binary at p value less than 0.05 were analyzed for multivariate logistic model using binary logistic analysis. Finally, variables which had significant association were identified on the basis of OR, with 95 % CI and p-value less than 0.05.

### Ethical consideration

The study was undertaken after the approval of the ethical committee of Debre Markos University and permission of Hawassa city administration education bureau were offered. Participation of all respondents was volunteer based. Measures were taken to assure respect, dignity and freedom of each individuals participating in the study. Information on the purpose and procedures of the study was explained. Confidentiality of information was assured verbally to all study subjects and informed consent assured before engaging into data collection.

## Results

### Socio demographic characteristics

This study had a response rate of 97.4 %. Out of the total 750 respondents, 386(51.5 %) were males, 489(65.2 %) from public school. 470(62.7 %) respondents were attending grade 11and the rest grade 12 students. The mean age of students was 18.14 with ±1.057 SD. From respondents, unmarried (single) respondents accounted 713 (95.1 %) and 487(64.9 %) living with parents (Table 1).

**Table 1:** Socio-demographic characteristics of youths attending preparatory school in Hawassa, Southern Ethiopia, May 2014

Variables	Category	Number(*n* = 750)	Percent
Age	15–16	31	4.1
	17–18	460	64.3
	19–24	259	34.5
Sex	Male	386	51.5
	Female	364	48.5
Religiosity	Attend frequently	485	64.7
	Attend sometimes	218	29.1
	Rarely	47	6.3
Marital status	Never married	713	95.1
	Married	37	4.9
Living arrangement	Both parents	487	64.9
	Relatives	101	13.5
	^b^Others	162	21.7
Fathers education	Illiterate, read and write	137	18.3
	Elementary	107	14.3
	Secondary	102	13.6
	Tertiary	404	53.9
Mothers Education	Illiterate, read and write	160	21.3
	Elementary	181	24.1
	Secondary	110	14.7
	Tertiary	299	39.9
Fathers occupation	Daily labourer	17	2.3
	Private employee	331	44.1
	Civil servant	321	42.8
	Does not work	70	9.4
	^c^Others	11	1.4
Mothers occupation	Daily laborer	12	1.6
	Private employee	376	50.1
	Civil servant	192	25.6
	Does not work	152	19.9
	^c^Others	18	2.4

N.B ^a^father only, lonely, friends, mother only, grandparents, Brother/sister, ^b^retired, consultant, student

**Table 2:** Exposure of respondents to sexually explicit reading materials among preparatory school youths of Hawassa city, May 2014

Variables	Category	Number	Per cent
Exposure to sexually explicit text(*n* = 750)	Yes	554	73.9
	No	196	26.1
Sources of sexually explicit text(*n* = 554)	Buying	75	13.5
	From school	84	15.2
	From friends	384	69.3
	From internet access	163	29.4
	others	5	0.9
Usual reading partner(*n* = 554)	Alone	384	69.3
	Same sex	103	18.6
	Opposite sex	32	5.8
	Family member	12	2.2
	Others	3	0.5
Frequency of reading(*n* = 554)	Once/twice per week	105	18.9
	Sometimes	442	78.9
	Often	7	1.3
HIV/FP usually mentioned (*n* = 554)	Yes	317	57.2
	No	237	42.8

### Substance use of respondents

**Fig. 2:**
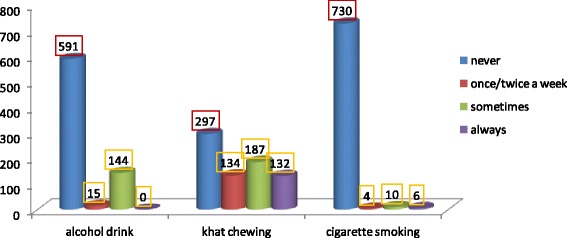
Frequency distribution of Substance use by respondents in preparatory school youths in Hawassa city, May 20014. N.B: Others include helping families, attending night club and religious ceremonies, and playing sport

About 591(78.8 %) respondents have never drank alcohol, 730(97.3 %) never smoked cigarettes and 297(39.6 %) never chewed *Khat*. Among the respondents who had labelled ‘some times’ in each variable, majority 187(24.9 %) were for chewing khat and few 10(1.3 %) cigarette smoking Fig 2.

### Spending leisure time

**Fig. 3:**
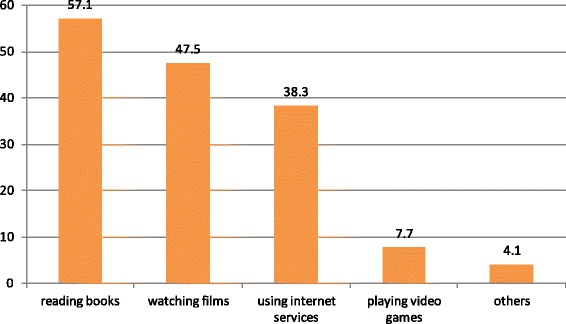
Per cent of respondents passing leisure time in preparatory school of Hawassa city, May 2014. N.B: others include school compound film show, friend’s home, and buying VCD player pornography

About 356(47.5 %) respondents were watching movies/TV shows, 287(38.3 %) spent by searching internet services, and 31(4.1 %) others (such as, sport and helping family) Fig. 3.

### Magnitude of exposure to SEMs

From the total respondents, around 579(77.2 %) were exposed for sexually explicit materials. Sex films with DVD player video television were the major source of sexually explicit material (64.0 %), followed by internet access (53.2 %) and mobile phone (41.6 %). Access to SEM was labeled ‘ easy’ by 484(64.5 %) from 750 respondents who participated.

Responding to the question of exposure to sexually explicit reading materials, 554(73.9 %) of the participants recalled their being exposed to such texts. Friends were the major source of the reading materials for 384(51.2 %). Internet access for sex oriented reading materials also had a considerable share (21.7 %).

Reading materials (texts) with high sexual content were usually read alone accounted 384(46.4 %) of the respondents, sharing with same sex friends was 103(13.7 %) respondents and with opposite sex friends by 32(4.3 %). With regard to frequency of reading, about 105(18.9 %) respondents read such materials rarely (once or twice) and 442 (79.8 %) read some times (Table 2).

Regarding exposure to sexually explicit films, 566(75.5 %) of the 750 respondents reported exposure. Among those who responded for how frequent, 15(2.7 %) reported watching sex films often, 503(88.9 %) sometimes and 48(8.5 %) once or twice. Internet searching was the major source of sexually explicit movies (45.9 %), followed by sharing by mobile phone Bluetooth among friends (36 %) and sharing from friends accounts (27.2 %). Other infrequently cited sources were rental, school and buying of such films by (22.4 %) respondents. Among the respondents who admitted of being exposed to SE films, about 219(38.7 %) reported of having exercised what they have seen in movies. Also, 142(25.1 %) exposed respondents had had sex after exposure and 30(5.3 %) experienced advanced sexual activities (such as, anal or oral). Majority of respondents reported that few films showed practice of safe sex (Table 3).

**Table 3:** Exposure of respondents to sexually explicit films in preparatory school youths of Hawassa city, May 2014

Variables	Category	Number	Percent
Exposure to sexually explicit films(*n* = 750)	Yes	566	75.2
	No	186	24.8
Sources of sexually explicit films(*n* = 566)^a^	Rental	80	14.1
	From friends	154	27.2
	From school	10	1.8
	Buying	20	3.5
	From internet search	260	45.9
	Mobile bluetooth	204	36.0
	Others	03	0.05
Usual viewing partner(*n* = 566)	Alone	274	48.4
	Same sex	226	39.9
	Opposite sex	35	6.2
	Others	12	2.1
Frequency of watching(*n* = 566)	Once/twice per week	48	8.5
	Sometimes	503	88.9
	Often	15	2.6
HIV/FP usually mentioned (*n* = 566)	Yes	61	10.8
	No	505	89.2
Tried what they have seen based on the films	Yes	219	38.7
	No	347	61.3
Played sex after exposure	Yes	142	25.1
	No	424	74.9
Played advanced sex after exposure	Yes	30	5.3
	No	536	94.5

^a^Multiple response

### Attitude towards sexually explicit materials

Out of 750 respondents, around 385 (51.3 %) had positive attitude towards the existence of SEMs while 365(48.7 %) had negative attitude to the presence of such materials. Around 348 (46.4 %) believed that SEM is able to change sexual behavior, while 290 (38.7 %) did not agree. 645 wished to learn the benefits and harm of being exposed to such materials either from their teachers or from their family (Table 4).

**Table 4:** Attitude of the respondents towards SEMs in preparatory schools of Hawassa city, May 2014

Variables	Category	Number	Percent
Existence of SEM important	Strongly agree	241	32.1
	Agree	234	31.2
	Neutral	72	9.6
	Disagree	105	14.0
	Strongly disagree	98	13.1
SEM exposure changes sexual behaviour	Strongly agree	167	22.3
	Agree	181	24.1
	Neutral	112	14.9
	Disagree	168	22.4
	Strongly disagree	122	16.3
Family/teacher should inform	Strongly agree	472	62.9
	Agree	173	23.1
	Neutral	35	4.7
	Disagree	30	4.0
	Strongly disagree	40	5.3

### Sources of information and accessibility of sexually explicit materials

**Fig. 4:**
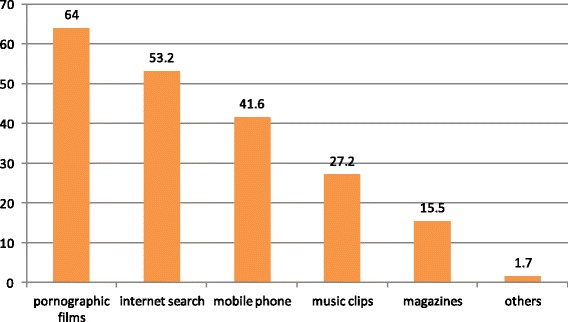
Source for exposure to SEM in preparatory school youths of Hawassa city by percent, May 2014

The major sources of information to preparatory youths on sexual issues were their friends (63.2 %). Among the respondents, about 522(70.4 %) claimed as having no open discussion on sexual issues within their family. Furthermore, About 450 (60.0 %) respondents said that they had received no sexual and reproductive Health education at school Fig. 4 and Table 5.

**Table 5:** Responses of respondents regarding sexual information in preparatory school youths of Hawassa city, May 2014

Variables	Category	Number	Per cent
Source of sexual information(*n* = 750)	From my family	245	32.7
	Other family members	130	17.3
	Sexual partner	210	28.0
	Friends/peers	474	63.2
	School	255	34.0
	Health institution	442	58.9
	Religious institution	234	31.2
	Mass media	403	53.7
Best source of information(*n* = 750)	My parents	111	14.8
	Other family members	10	1.3
	Sexual partner	66	8.8
	Friends/peers	144	19.0
	School	56	7.5
	Health institution	147	19.6
	Religious institution	84	11.2
	Mass media	129	17.2
Open discussion with family members (*n* = 750)	Yes	222	29.6
	No	528	70.4
SRH education at school (*n* = 750)	Yes	300	40.0
	No	450	60.0

### Factors associated with exposure to SEM

The multivariate logistic regression analysis observed that being a male student had shown two times higher exposure to SEM than being a female (95 % CI: COR, 2.16(CI = 1.52, 3.07). A Student who attended private schools had almost two times higher exposure to SEM (95 % CI: COR 1.67(CI = 1.14, 2.43) than students who attended public schools (Table 6).

**Table 6:** Factors showing over all exposure and association to SEM among preparatory schools youth Hawassa city, May 2014

Variables	Category	Frequency of SEM exposure		COR(95 % C.I)	AOR^a^(95 % C.I)
		Yes	No		
SEX	Male	323	63	2.16(1.52,3.07)^b^	1.844(1.22,2.78)^b^
	Female	256	108	1.00	1.00
School type	Private	216	45	1.67(1.14,2.43)^b^	2.07(1.29,3.30)^b^
	Public	363	126	1.00	1.00
Living condition	Both parents	357	130	1.00	1.00
	Mother	43	4	3.91(1.38,11.2)^b^	3.03(0.93,9.84)
	Grand parents	86	15	2.08(1.16,3.74)^b^	1.77(0.89,3.52)
	Relatives	78	18	1.58(0.91,2.74)	1.05(0.54,2.03)
	Others	15	4	1.36(0.44,4.19)	0.95(0.27,3.29)
Father education	Illiterate, read and write	121	16	2.66(1.52,4.74)^b^	1.67(0.80,3.48)
	Elementary	81	26	1.10(0.67,1.81)	0.86(0.42,1.74)
	Secondary	79	23	1.22(0.73,2.04)	1.21(0.62,2.37)
	Tertiary	298	106	1.00	1.00
Mother education	Illiterate, read and write	136	24	1.96(1.18,3.25)^b^	1.39(0.67,2.89)
	Elementary	137	44	1.08(0.70,1.65)	1.09(0.59,2.01)
	Secondary	84	26	1.12(0.67,1.86)	0.89(0.48,1.66)
	Tertiary	222	77	1.00	1.00
Alcohol drinking	Never	436	155	1.00	1.00
	Sometimes	143	16	3.18(1.83,5.49)^b^	2.33(1.26,4.31)^b^
Khat chewing	Never	188	109	1.00	1.00
	Once/twice	113	21	3.12(1.85,5.25)^b^	3.02(1.65,5.52)^b^
	Sometimes	166	21	4.58(2.74,7.64)^b^	3.40(1.93,6.00)^b^
	Often	112	20	3.24(1.90,5.52)8	2.67(1.46,4.86)^b^
Possibility of getting SE Films	Easily	434	50	6.62(4.33,10.14)^b^	5.64(3.56,8.94)^b^
	Difficult	52	50	0.79(6.48,1.30)	0.59(0.34,1.05)
	Impossible	93	71	1.00	1.00

^a^AOR: adjusted for sex, school type, alcohol drinking, *khat* chewing, possibility of getting SEM

^b^statistically Significant, 1.00 --- constant

Students who were those living with mother only revealed four times greater exposure to SEM than living with both biological parents (95 % CI: COR 3.91(CI = 1.38, 11.12) and those living with grandparents also revealed two times higher exposure (95 % CI: COR 2.08(CI = 1.16, 3.74) to SEM. Regressing the educational status of mother and father, those students whose fathers could not read and write were three times more exposed than those whose fathers had obtained tertiary education (95 % CI: COR 2.69(CI = 1.52, 4.47). Students whose mother could not read and write were two times more exposure than students whose mothers attended tertiary education (95 % CI: COR of 1.96(CI = 1.18, 3.25) to SEM (Table 6).

Students who were taking alcohol labelled ‘sometimes’ had three times greater exposure to SEM than those not taking alcohol (95 % CI: COR 3.18(CI = 1.83, 5.49). Those students who had Chewed small case (such as, rarely) showed three times increased exposure (95 % CI: COR 3.12(1.85, 5.25), labelled ‘sometimes’ were five times higher exposure (95 % CI: COR 4.58(2.75, 7.64), and labelled ‘often’ revealed three times greater exposure (95 % CI: COR 3.45(1.90, 5.52) to sexually explicit materials. Finally, the possibility of getting SEMs labelled ‘Easy access’ shown by odds of seven folds (95 % CI: COR of 6.63(CI = 4.33, 10.14) exposed to SEM (Table 6).

## Discussion

This study attempted to assess the magnitude of exposure to SEMs and factors associated in preparatory youths’ in Hawassa city, Southern Ethiopia. Accordingly, about 77.2 % of respondents had been exposed to SEMs. Experience of exposure to SEM in this study was greater than in previous studies conducted in Addis Ababa [20]. The difference might be due to difference in prevalence of problems by regions and the difference in preventive health service activities.

In this study, internet searching was the major source of information for sexually explicit materials/ movies (45.93 %) followed by sharing by mobile phone Bluetooth among friends (36.04 %). But, in Addis Ababa study, video rental was a major source. In case of text exposure, friends were the main sources to SEM [20]. Currently this change might be due to increased access to portable SEM/media and internet services in the country and in the fastest growing city, Hawassa.

This study revealed that more than 70 % of adolescents had no discussion on sexual issues with their parents. The majority of parents never control what their adolescents are doing and where they are. A previous study showed 55 % of respondents had no sexual discussion at home [20]. This difference may be due to difference in cultural and developmental status difference in both studies.

This study showed that about 60 % of respondents reported they did not have sexual and reproductive health education at school. This was more than the findings of a study in Addis Ababa study in 2008 (60 % VS 43.6 %) [18]. This difference might be due to low discussion on sexual issues at Hawassa by student’s family and School Reproductive Health education at school.

This study had found that respondents who exposed to SEMs experienced risky sexual behaviours. Around 38.7 % tried to do what they had seen in SEM, 25.08 % played sex after exposure and 5.3 % did sexual activities like anal or oral sex. Similar findings were observed in different studies outside home [9–11]. This could show that exposure to SEM may have relation to risky sexual behaviour in the areas of study’s findings.

Unwanted solicitation to sexually explicit media and internet contents was reported by 32.8 % of the respondents in this study. This was nearly similar with findings of previous home study (32.8 % VS 27 %) [20] and lower from findings of New Hampshire State(USA) National mobile based survey (32.8 % VS 52.5 %) [4]. Similar finding could be due to more or less similar level of access to internet across the country. As compared to the American study, lower findings in Ethiopia could be related to lower access, coverage and/or skill for utilization of internet and the vice versa in U.S.A.

The multivariate analysis carried out using binary logistic regression indicated that being male students had almost 1.8 times higher exposure to SEMs when compared to female students (95 % CI: AOR 1.84(C.I = 1.22, 2.79). It was concurrent with studies done elsewhere [3, 5, 7]. This similarity could be due to culture contribution for better access of male students to SEM/media in all the study areas.

Those students who attended private schools were significantly associated with exposure to SEM (AOR = 2.07; 95 % CI: 1.29, 3.30). This significant difference might be due to students in private schools had better income to access internet services and modern SEM/media. It was inconsistent with previous study done in the home land(Addis Ababa) [19] in that the capital of Ethiopia could have more free or low price internet access as compared to Hawassa. This makes equal opportunity internet access to private (such as, rich family) and government (such as, poor family) school youths.

The multivariate analysis on substance use showed that students who drinks alcohol sometimes showed significant association to SEM than students who never drink alcohol (AOR = 2.33; 95 % CI: 1.26, 4.30) and it was supplemented by other study done at home [12]. Khat chewing among respondents had also found to be independent factor for exposure to SEM. Students who chew Khat were highly exposed to SEMs in all categories of chewers labeled from ‘rarely(once/twice per week), (AOR 3.02, 95 % CI:1.65,5.52), labeled ‘sometimes’ with (AOR = 3.40, 95 % CI: 1.93,6.00) to ‘often’ with (AOR = 2.67, 95 % CI: 1.46,4.86). This significant association might also be due to increased alcohol and Khat chewing houses around and nearby schools compound. These associations were not in line with a previous study done in Addis Ababa in 2008 [19]. This could be due to low incidence and prevalence of alcohol and khat users among previous youths as compared to this current generation youths.

Possibility of getting SEM among students reported by majority that they can access easily. It had got almost odds of six folds of exposure by students labeled ‘easy access with (95 % CI: AOR 5.64(CI = 3.56, 8.94) than with no access. This might be due to increased portability of laptops, cell phones and other modern SEM media in our country. Minimizing opportunities to access SEM and/or discussing risks after exposure to SEM among students was the way forwarded by this study.

## Conclusion and recommendations

This study found that high numbers of students were exposed to sexually explicit materials. School youths were often exposed to SEM within their immediate environment through friends and family members. Sex, school type, substance use and access towards SEM were observed as independent predictors for exposure to SEM in this study. The government, especially MOH and MOE should adopt regulatory strategies to minimize the harms associated with young people’s exposure to sexually explicit content through mass media and internet access. The mass media should play powerful role in the socialization of school adolescents and in shaping young people’s sexual knowledge, attitudes, and behaviors. The Hawassa city Health and education bureau should offer basic and refresher training for teachers and staff members on school health, mini media clubs at school so as to decrease opportunities for exposure to SEM. The health facilities should do health promotion and awareness creation regarding substance use and sexual and reproductive health for all clients on a regular basis.

## Abbreviations

SD: Standard deviation
SEM: Sexually explicit material
AOR: Adjusted odds ratio
MOH: Ministry of Health
MOE: Ministry of Education
SE: Sexually explicit
